# Breastfeeding performance index and associated factors among children aged 0–6 months in Ethiopia: Analysis of the 2019 Ethiopia Mini Demographic and Health Survey

**DOI:** 10.3389/fnut.2022.970737

**Published:** 2022-10-03

**Authors:** Getachew Tilahun Gessese, Berhanu Teshome Woldeamanuel, Takele Gezahegn Demie, Tolesa Diriba Biratu, Simegnew Handebo

**Affiliations:** ^1^Department of Nutrition, School of Public Health, St. Paul's Hospital Millennium Medical College, Addis Ababa, Ethiopia; ^2^Department of Epidemiology and Biostatistics, School of Public Health, St. Paul's Hospital Millennium Medical College, Addis Ababa, Ethiopia; ^3^Department of Health Education and Health Promotion, School of Public Health, St. Paul's Hospital Millennium Medical College, Addis Ababa, Ethiopia

**Keywords:** breastfeeding, breastfeeding performance index, infant feeding, breast milk, Ethiopia

## Abstract

**Background:**

Infants under the age of 6 months are commonly affected by malnutrition globally. The higher the breastfeeding performance index (BPI), the greater the advantage of breastfeeding will be. However, there is a lack of literature in the context of Ethiopia. Therefore, this study is aimed at investigating the magnitude and determinants of the breastfeeding performance index score among mothers of children under the age of 6 months in Ethiopia.

**Methods:**

This study was conducted using the 2019 Ethiopia Mini Demographic and Health Survey (EMDHS) dataset. A stratified, two-stage cluster sampling technique was used in the study. The survey data were weighted using the “svy” function in STATA version 16. Descriptive statistics, bivariable and multivariable logistic regression were employed in the analysis. An adjusted odds ratio (*AOR*) with a 95% confidence interval (*CI*) was reported. The results were considered statistically significant if the *p*-value was < 0.05. The goodness of fit of the model was checked using the Hosmer–Lemeshow test.

**Results:**

A total of 4,273 mothers with children under the age of 6 months were included in the analysis. Our analysis revealed that the prevalence of low breastfeeding performance index was 79.05% (95% *CI*: 78.01, 81.59). A unit increase in child age (*AOR* = 11.56; 95% *CI*: 6.97, 19.17), the richest wealth quintile (*AOR* = 2.76; 95% *CI*: 1.18, 6.5), a higher level of education (*AOR* = 5.41; 95% *CI*: 2.08, 14.05), being married or living with partner (*AOR* = 2.73; 95% *CI*: 1.18, 6.27), being women from Somali (*AOR* = 5.11; 95% *CI*: 2.08, 12.56), Afar (*AOR* = 3.03; 95% *CI*: 1.16, 7.91), Oromia (*AOR* = 1.88; 95% *CI*: 1.03, 3.41), Diredawa city administration (*AOR* = 2.89; 95% *CI*: 1.04, 8.07), and antenatal care (ANC) visit (*AOR* = 2.05; 95% *CI*: 1.31, 3.19) were positively associated with the low breastfeeding performance index.

**Conclusion:**

The prevalence of the low breastfeeding performance index was found to be high. Hence, the findings of the study suggest the need to target interventions aimed at improving breastfeeding performance toward mothers with higher socioeconomic and demographic status and educational status. Antenatal care clients are among the targets of the intervention.

## Introduction

Many developing countries are affected by malnutrition, which has serious ramifications for individual health and national development in terms of lost human capital and economic output (1–4). Infants under the age of 6 months are commonly affected by malnutrition globally (4). The third Copenhagen Consensus in 2012 highlighted nutrition as the best investment for poor countries, with every dollar invested yielding a US$ 30 return (5). As optimal breastfeeding is one of the recommended nutrition interventions among infants and young children and contains various subcomponents (6, 7), the breastfeeding performance index (BPI) therefore attempts to synthesize key elements of optimal breastfeeding habits into a single summary variable by combining the many characteristics of breastfeeding practices (8).

The World Health Organization (WHO) defines optimal breastfeeding as the initiation of breastfeeding within 1 h of birth, exclusive breastfeeding for the first 6 months of life, and continuous breastfeeding for up to 2 years or beyond, with appropriate complementary feeding starting at 6 months (5, 9).

Adhering to all three WHO recommendations is crucial to lowering both newborn and child mortality as breastfeeding protects against illness and aids the recovery of sick children (10, 11). This is because for newborns, breast milk is the best food. It is safe, hygienic, and includes antibodies that help protect children against a variety of ailments. Breastmilk provides all of the energy and nutrients that an infant requires during the first few months of life, and it continues to provide up to half or more of a child's nutritional needs during the second half of the 1st year and up to one-third of a child's nutritional needs during the 2nd year (12). Therefore, optimal breastfeeding reduces the risk of child mortality (10, 13–17). In addition, children who are breastfed score higher on intelligence quotient (IQ) tests, are less likely to be overweight or obese, and are less likely to develop diabetes later in life (9, 12, 18).

Except for a few rare medical conditions specified by the WHO and UNICEF, exclusive breastfeeding from birth is possible, and virtually every mother can breastfeed (19). Compared with exclusively breastfed newborns, infants who were predominantly, partially, or not breastfed had a significantly higher risk of all-cause and infection-related mortality (10). Therefore, to promote the best growth, development, and health, infants should be breastfed exclusively for the first 6 months of life (20).

However, breastfeeding duration is shorter in high-income countries than in low and middle-income ones. Even in low- and middle-income nations, only 37% of infants younger than 6 months are breastfed exclusively (21). Nearly two out of every three infants are not exclusively breastfed for the recommended 6 months, a rate that has remained unchanged for the past two decades (12), though it is known that mothers should practice exclusive breastfeeding (22).

The national Infant and Young Child Feeding (IYCF) Strategy of Ethiopia recommends exclusive breastfeeding as one of the components in its guide to infant and young child feeding practice (23), and has been implemented over decades. However, in 2019, only 59% of children under the age of 6 months are exclusively breastfed. In a similar year, 72% of newborns were breastfed within 1 h of birth, declining from 73% in 2016; and 12% of children received a prelacteal food, increasing from 8% in 2016. Furthermore, contrary to the recommendation that children under 6 months be exclusively breastfed, 14% of infants also received water, 1% received non-milk liquids, such as juices and clear broth, and another 8% received other milks before reaching the age of 6 months (24).

Mixed feeding is one of the global challenges, having its own risks to health and child survival. According to Monge-Montero C. et al.'s systematic review and meta-analysis, the overall prevalence of mixed milk feeding varied between 23 and 32 across different age intervals and geographies around the world; the highest rate was identified for the age group of 4–6 months (25). In addition, a study revealed its impact on child health, indicating the number of episodes of bronchiolitis in infants with exclusive breastfeeding and mixed feeding was reduced by 41 and 37%, respectively, compared with infants who did not breastfeed. This indicates that though mixed feeding is better than not breastfeeding at all, switching from exclusive breastfeeding to mixed feeding increased the incidence of bronchiolitis (26).

As a result, the higher the breastfeeding performance index, the greater the advantage of breastfeeding will be. Senarath U et al. found that assessing breastfeeding practice using the breastfeeding performance index was both feasible and beneficial for intervention (27).

According to the scant literature on the subject available in Ethiopia, the prevalence of low, medium, and high BPI was 18.41, 57.96, and 23.63%, respectively (8). Another recent study by Hailu WS et al. indicated that the prevalence of low breastfeeding performance index was 40.7% in the north-west part of Ethiopia (28).

Various factors were found to have an effect on the low breastfeeding performance index. According to Hailu WS et al., occupation of the mother, marital status, antenatal visits, postnatal care follow-up, attitudes of mother toward breastfeeding, and breast-feeding knowledge of the mother, were among the determinates of the BPI score (28).

Given the scarcity of recent evidence on the breastfeeding performance index in the country in general and with regard to what factors contribute to the determinants of the breastfeeding performance index in particular. Therefore, the objectives of this analysis were to determine the prevalence of the breastfeeding performance index and identify factors affecting it to guide evidence-based interventions and policy directives.

## Methods

### Study design

With permission of the Demographic and Health Survey (DHS) program, a secondary data analysis was performed using the 2019 EMDHS extracted from http://www.DHSprogram.com. The Ethiopian Demographic and Health Survey (EDHS) was undertaken in nine Ethiopian regions (Tigray, Afar, Amhara, Oromia, Somali, Benishangul Gumuz, the Southern Nations Nationalities and Peoples (SNNP), which currently includes Sidama and Southwestern Ethiopia regions, Gambella, and Harari) and two chartered city administrations (Addis Ababa and Dire Dawa) from 21 March 2019 to 28 June 2019. The study design was cross-sectional and is described in detail in the Ethiopia Mini Demographic and Health Survey (EMDHS) 2019 (29).

### Sampling technique and study population

The sample of the 2019 Demographic and Health Survey (EDHS) was designed to provide data at the national (urban and rural) and regional levels. A stratified, two-stage cluster sampling technique was used in the study. Enumeration areas (EAs) were the sampling units for the first stage of the 2019 EDHS sample, where 305 of them were randomly chosen. In the second stage, a representative sample of 8,794 households with 9,012 reproductive-age women who gave birth in the last 5 years was chosen. However, this study's analysis was limited to 4,273 women with infants aged 0–6 months (29).

### Data extraction

The Ethiopian DHS 2019 data were downloaded in STATA format from the Measure DHS website. We explored and coded the data after having a better knowledge of the details. The final analysis used data with information on a wide range of prospective variables, such as socio-demographic traits, economic variables, and breastfeeding patterns.

### Study variables

The outcome variable was the breastfeeding performance index (BPI) among 0–6-month old children. The BPI was calculated by assigning one point to each of seven infant feeding practices: first suckling within an hour of birth; absence of pre-lacteals; non-use of feeding bottles; current breastfeeding; not receiving liquids, formula, or other milk; and not receiving solids in the previous 24 h (**Table 2**). A low breastfeeding performance index score was declared for women who did not practice one or more of the breastfeeding performance index scoring variables according to the recommended standard, leading to partial breastfeeding practice. Therefore, women with ≤6 breastfeeding performance scores were categorized as having a low BPI, whereas women with a breastfeeding performance score of 7 were considered as having a high BPI. The independent variables in the study were: child characteristics, such as sex and age; mother's characteristics, such as age, level of education, marital status, religion, region, place of residence, use of ANC, place of delivery, cesarean section delivery, post-natal care, and the household wealth index were used to estimate the household's economic status.

### Statistical analysis

STATA version 12 was used to analyze the data. The survey data were weighted using the “svy” function in STATA version 16. Descriptive statistics in the form of frequencies and percentages with confidence intervals (*CI*s) were used to describe the study population in relation to independent variables. The association between the breastfeeding performance index and independent variables was analyzed using binary and multivariable logistic regression models. Variables with a statistically significant association with BPI in bivariable logistic regression (*p*-value 0.2) were considered for inclusion in the final multivariable model. In the final multivariable logistic regression model, the relationship of BPI with independent variables was considered significant at a *p*-value of < 0.05. Both crude odds ratios (*CORs*) and adjusted odds ratios (*AOR*s) were reported with 95% confidence intervals. The goodness of fit of the model was checked using the Hosmer–Lemeshow test (*F* = 0.25). Tables and graphs were used to summarize the findings.

## Results

### Socio-demographic and reproductive health characteristics of the respondents

A nearly equal proportion (one in every five) of respondents came from one of the five wealth index categories. Almost half (51.6%) of women did not have any exposure to education, while more than one-third (36%) had only a primary level of education. The vast majority (93.2%) of women were married or living with their partners, and nearly 90% of them were from any of the three bigger regions; Oromia, Amhara, and SNNP regions. About 43.5% of women attended antenatal care (ANC) four or more times and nearly a quarter of them, 24.1 and 23.3%, respectively, had infants aged 0– and 1–month old (Table 1).

**Table 1:** Socio-demographic and reproductive health characteristics of women and children enrolled in the analysis of the breastfeeding performance index (BPI), the 2019 Ethiopia Mini Demographic and Health Survey (EMDHS) (*n* = 4,273).

**Variables**		**Weighted frequency**	**Weighted percentage**
Wealth index	Poorest	880	20.60
	Poor	883	20.68
	Middle	838	19.61
	Richer	800	18.71
	Richest	872	20.40
Region	Tigray	313	7.40
	Afar	53	1.25
	Amhara	892	21.08
	Oromia	1623	38.39
	Somali	218	5.16
	Benishangul Gumuz	49	1.17
	SNNPR^*^	886	20.95
	Gambella	21	0.5
	Harari	12	0.29
	Addis Ababa	138	3.27
	Diredawa	23	0.55
Place of residence	Urban	1120	26.23
	Rural	3152	73.77
Educational status of the mother	No education	2203	51.57
	Primary	1538	36.0
	Secondary	360	8.42
	Higher	171	4.01
Sex of the child	Male	2257	52.84
	Female	2015	47.16
Age of the child in months	0 month	1029	24.08
	1 month	996	23.32
	2 months	800	18.72
	3 months	564	13.21
	4 months	382	8.94
	5 months	245	5.72
	6 months	257	6.02
Age category of the mother	<20	209	4.89
	20-34	2964	69.37
	≥35	1099	25.73
Religion	Orthodox	1573	36.82
	Protestant	1234	28.90
	Muslim	1392	32.59
	Others^**^	72	1.70
Marital status	Married or living with partner	3983	93.23
	Single or living alone	289	6.77
Antenatal care visits	Not attended	944	25.03
	1–3 times visits	1185	31.43
	Four and more visits	1642	43.54
Place of delivery	Home	1788	47.91
	Health facility	1944	52.09
Cesarean section delivery	No	3544	93.99
	Yes	227	6.01
Postnatal visit	No	3248	86.15
	Yes	522	13.85

* South Nations Nationalities and People's Region.

** includes Catholic, Traditional, and others.

### Breastfeeding performance index

The breastfeeding performance index (BPI) scoring system for infants 0–6 months included seven components (Table 2). The minimum BPI score was 0 while the maximum was 7. The mean score and standard deviation (SD) for the breastfeeding performance index were 5.15 (1.39).

**Table 2:** The breastfeeding performance index components and the scoring system among infants aged 0–6 months, Ethiopia, 2019.

**Feeding practices**		**Weighted frequency**	**Weighted percentage**	**score**
Timely initiation of breastfeeding	≤1 hr	2740	75.83	1
	>1 hr	873	24.17	0
Pre-lacteal feeding	Yes	375	10.38	0
	No	3239	89.62	1
Current breastfeeding	Yes	1305	64.95	1
	No	704	35.05	0
Liquids given	Yes	541	26.92	0
	No	1469	73.08	1
Bottle feeding	Yes	639	16.94	0
	No	3132	83.06	1
Solid food given	Yes	1305	64.95	0
	No	704	35.05	1
Formula milk given	Yes	78	3.88	0
	No	1932	96.12	1

About a quarter (14.17%) of the respondents had no timely initiation of breastfeeding, and nearly one in ten respondents (10.38%) practiced prelacteal feeding. Nearly two-thirds (64.95) of the respondents were breastfed in the last 24 h (Table 2).

**Figure 1:**
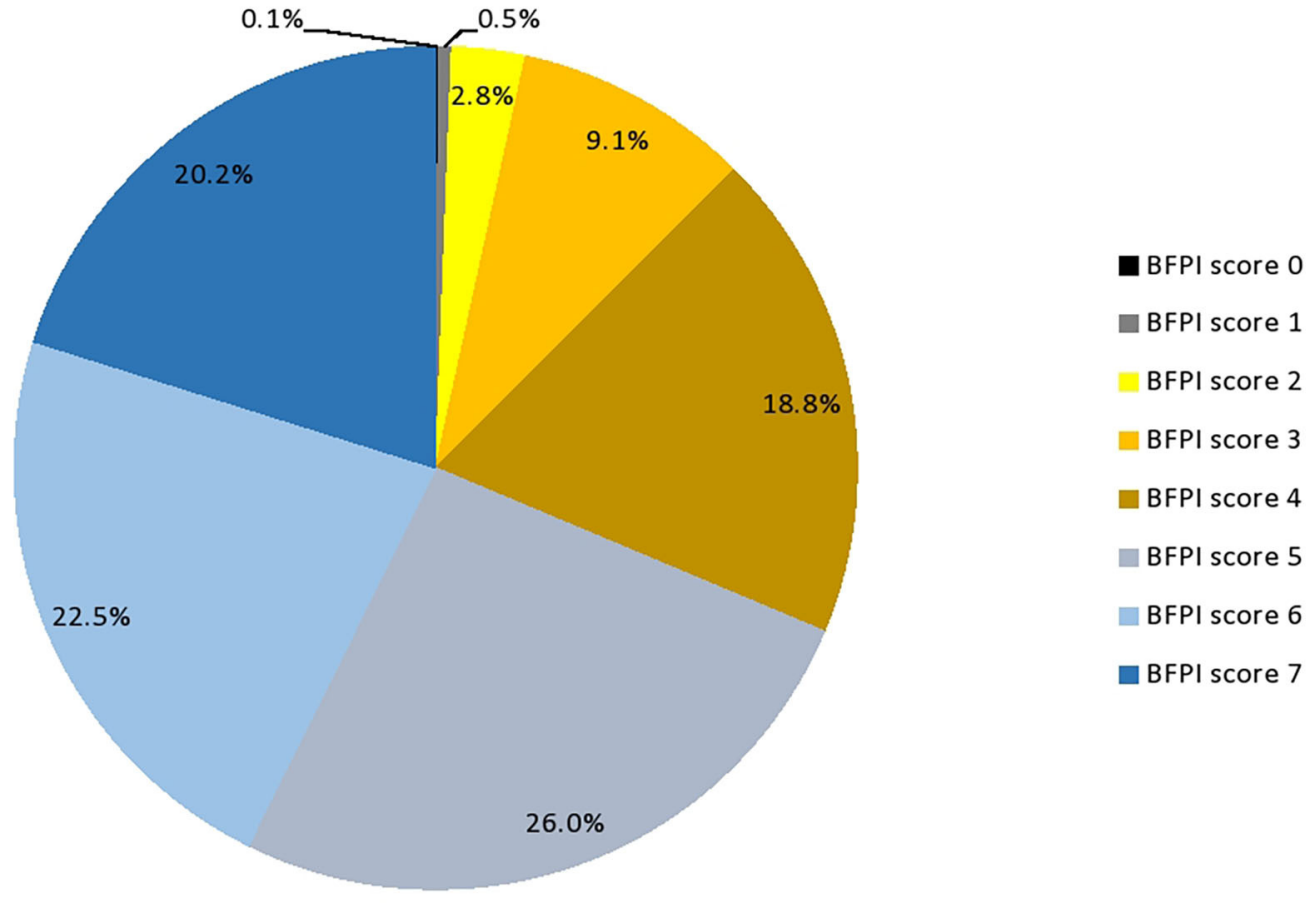
Breastfeeding performance score of reproductive age mothers in Ethiopia.

The prevalence of the low breastfeeding performance index was 79.05% (95% *CI*: 78.01, 81.59). This implies that only one out of five breastfeeding mothers with children under the age of 6 months correctly performs the recommended breastfeeding practice (Figure 1).

### Factors associated with low breastfeeding performance index

In the final multivariable model adjusted for the potential confounders, the BPI was significantly associated with age of the child, wealth index, educational status, marital status, region of residence, and number of antenatal care visits.

A 1-month increase in child age increases the odds of low breastfeeding performance by eleven times (*AOR* = 11.56; 95% *CI* = 6.97, 19.17). The odds of having a low breastfeeding performance index were more than two and a half times higher in the richest wealth quintile than in the poorest (*AOR* = 2.76; 95% *CI*: 1.18, 6.5). In addition, the low breastfeeding performance index was more than five times higher among women with a higher level of education than among women with no education (*AOR* = 5.41; 95% *CI*: 2.08, 14.05). Being married or living with a partner increases the odds of low breastfeeding performance (*AOR* = 2.73; 95% *CI*: 1.18, 6.27) as compared with singles or living alone due to divorce, widowed, or any other reason. Compared with women from the Tigray region, women from Somali (*AOR* = 5.11; 95 % *CI*: 2.08, 12.56), Afar (*AOR* = 3.03; 95% *CI*: 1.16, 7.91), Oromia (*AOR* = 1.88; 95% *CI*: 1.03, 3.41) regions, and Diredawa city administration (*AOR* = 2.89; 95% *CI*: 1.04, 8.07) had higher odds of having a low breastfeeding performance index. Having 1–3 antenatal care visits during their index child's pregnancy was associated with two times higher odds of low breastfeeding performance index (*AOR* = 2.05; 95% *CI*: 1.31, 3.19) than women with no antenatal care visits (Table 3).

**Table 3:** Factors associated with the BPI in children aged 0–6 months in Ethiopia, the bivariate and multivariate multilevel logistic regression analysis (*n* = 1,985).

**Variables**		**Low BPI**	
		**COR** **(95% CI)**	**AOR** **(95% CI)**
Age of the child		10.85 (6.52, 18.08)	11.56 (6.97, 19.17)
Wealth index	Poorest	1	1
	Poor	1.19 (.71, 1.99)	1.46 (.79, 2.70)
	Middle	1.31 (.81, 2.12)	1.79 (.99, 3.24)
	Richer	1.32 (.79, 2.21)	1.73 (.91, 3.29)
	Richest	2.87 (1.62, 5.08)	2.76 (1.18, 6.50
Educational status of the mother	No education	1	1
	Primary	1.28 (.88, 1.86)	1.27 (.91, 1.79)
	Secondary	1.15 (.65, 2.02)	.99 (.49, 2.02)
	Higher	6.21 (2.59, 14.86)	5.41 (2.08, 14.05)
Antenatal care visits	Not attended	1	1
	1–3 times visits	1.94 (1.27, 2.97)	2.05 (1.31, 3.19)
	Four and more visits	1.72 (1.18, 2.51)	1.40 (.88, 2.23)
Marital status	Married or living with partner	2.15 (.99, 4.64)	2.73 (1.18, 6.27)
	Single or living alone	1	1
Region	Tigray	1	1
	Afar	1.35 (.64, 2.89)	3.03 (1.16, 7.91)
	Amhara	1.42 (.76, 2.67)	1.74 (.82, 3.68)
	Oromia	1.28 (.68, 2.41)	1.88 (1.03, 3.41)
	Somali	1.69 (.83, 3.41)	5.11 (2.08, 12.56)
	Benishangul Gumuz	.82 (.48, 1.41)	1.20 (.60, 2.42)
	SNNPR	.79 (.40, 1.55)	.98 (.49, 1.95)
	Gambella	.82 (.45, 1.50)	.78 (.31, 1.93)
	Harari	1.46 (.71, 3.01)	1.51 (.68, 3.36)
	Addis Ababa	1.83 (.90, 3.69)	1.03 (.42, 2.55)
	Diredawa	2.67 (1.18, 6.03)	2.89 (1.04, 8.07)

## Discussion

Breastfeeding, more than any other preventive measure, has the greatest potential influence on child health and mortality (30). Our analysis determined the breastfeeding performance of mothers having infants < 6 months of age by using the breastfeeding performance index, which comprises seven components, and identified factors contributing to low breastfeeding performance in Ethiopia from the 2019 Mini Demographic and Health Survey data of Ethiopia.

About four women out of five had a low breastfeeding performance index (79.05%). This indicates that the proportion of mothers with a high BPI was slightly lower in this study than the finding reported by Haile D. and Biadgilign S., where 23.6% of mothers had a high BPI (8). The difference may be related to the relatively higher cut-off point used to determine the low BPI category in our study that resulted in a low proportion of mothers with a high breastfeeding performance index.

Various maternal, child, and healthcare service-related factors were found to have affected the breastfeeding performance index. Our investigation revealed that child age, region of residence, household wealth quintile, antenatal care (ANC) contacts, educational status, and marital status as factors significantly associated with the breastfeeding performance index.

Child age is found to affect the breastfeeding performance index of mothers. For every 1-month increase in the child's age, mothers had an eleven times higher odds of low breastfeeding performance. Evidence indicates that as the age of the child increases, the probability of suboptimal breastfeeding increases (31–33). This could be related to mothers' tendency to substitute breastfeeding with other child feeding practices as the child's age advances. Substituting breastfeeding with other child feeding practices may be related to increased mothers' probability of getting back to work as the age of the child increases and maternity leave ends, leading mothers to cease breastfeeding early (32). Moreover, it may be related to shorter birth intervals and shorter duration of traditional postpartum care in Ethiopia by letting the mother stay in the home, which is for a maximum of 80 days (34).

In addition, economic status was among the factors impacting breastfeeding. Compared with women from the poorest wealth quintile category, the low breastfeeding performance index was more than two and half times higher among women from the richest wealth quintile category. Even though some findings indicate that mothers with a higher income status breastfeed better (33, 35), in contrast, there are findings indicating that mothers with a higher income category breastfeed less than those from low income categories (36, 37) and this supports the finding in our study. This could occur because mothers with higher income levels can afford infant formula and thus easily substitute breastfeeding with it while focusing on other life activities. In addition, another study claimed that a lack of workplace breastfeeding laws, support, and arrangements may interfere with the breastfeeding practice of employed mothers (38) with a better income status.

Furthermore, having a higher educational status increased the odds of low breastfeeding performance among mothers. It was more than five times higher than those women with no education. Despite studies indicating a positive association between breastfeeding and education (39, 40), a study by Tang K. et al. supports our finding that mothers with higher educational status breastfeed less, particularly with regard to exclusive breastfeeding practice, as opposed to early initiation of breastfeeding (37). This may happen because their educational status enables mothers to understand the benefits of breastfeeding. On the other hand, women with a better educational status may be engaged in income-generating employment. Therefore, it may result in a lack of time to focus on breastfeeding practice as opposed to women with low educational status who tend to be housewives.

Unexpectedly, women who are married or have a partner living with them had an increased low breastfeeding performance index as compared with singles or those living alone due to divorce, widowed, or any other reason. However, similar findings were reported by studies in some parts of Ethiopia where single mothers breastfeed better than married mothers (33, 41). This might be related to both the income status and work situation of mothers. Married mothers and those who live with their partners may afford breast milk substitutes more than single mothers or those who live alone.

Our analysis revealed that having antenatal visits increased the odds of having a low breastfeeding performance index. Unlike the positive association between antenatal care services and breastfeeding practice reported elsewhere (42–44), findings in this nationally representative large-scale study may possibly indicate improper breastfeeding counseling to ANC attendant mothers. It may also be due to the selective accessibility of the antenatal service in the healthcare system of the country to those mothers who are wealthier, highly educated, and employed mothers with the capacity to afford breastfeeding substitutes and have less time freedom to breastfeed their children in line with the recommendation regardless of their breastfeeding literacy.

Regional differences were observed in the breastfeeding performance index. Compared with women from the Tigray region, women from Somali, Afar, and Oromia regions, and Diredawa city administration had Higher odds of having a low breastfeeding performance index. Similar inter-regional discrepancies were reported in Ethiopia (45), which can be explained by variations in socio-economics, culture, and healthcare service availability and accessibility across regions.

## Strength and limitations

One of the strengths of the study was that the analysis depends on a nationally representative survey with a large sample size that ensures high precision in the findings. In addition, the study dealt with a composite index, which enables us to determine the broader picture of breastfeeding practice, which otherwise could remain obscured within the individual practices that constitute the index and thus may mask the existence of important relationships between BPI and its determinants.

However, the limitation of this study is that we were unable to check the association between the low breastfeeding performance index and childhood illnesses due to lack of data on such variables. Though it is not possible to determine the cause-effect relationship in this regard due to the chicken egg dilemma in the nature of the study design, had the data been obtained and analyzed, it would have the benefit of exposing the possible link between the BPI and childhood illness.

## Conclusion

We found that four out of five mothers do not fulfill the optimal exclusive breast breastfeeding practice. Child age, antenatal care service, and maternal socio-economic and demographic factors affect the breastfeeding performance of women. However, the analysis revealed the rarely reported relationships between breastfeeding practice and its determinants. The direction of the relationship between factors with the breastfeeding performance index was found to be mostly inversed and against commonly supported scientific insights that are reported when the breastfeeding performance index score subcomponent practices are analyzed separately. Our findings suggest that factors determining breastfeeding might have shifted toward an unusual direction of relationship with the breastfeeding performance of mothers. Therefore, in addition to redirecting the usual focus of intervention targets by eliminating the usual perception that better socio-economic, demographic, and service factors result in better breastfeeding practices, it also implies the need to validate findings with further longitudinal investigations in the local context to strengthen evidence and re-orient the policy interventions.

## Data availability statement

Publicly available datasets were analyzed in this study. This data can be found here: http://www.DHSprogram.com.

## Ethics statement

The study used data from the 2019 EMDHS obtained from Measure DHS data archive http://www.DHSprogram.com, with the appropriate request and permission. The DHS program owns data that are collected following all the necessary ethical procedures in accordance with the relevant guidelines and regulations. Therefore, for the data used in this analysis, all methods were carried out in accordance with relevant guidelines and regulations. The DHS Program is authorized to distribute, at no cost, unrestricted survey data files for legitimate academic research. Registration is required for access to data. The patients/participants provided their written informed consent to participate in this study.

## Author contributions

GTG conceived the study design. GTG, BTW, TGD, SH, and TDB carried out the statistical analysis and conducted the literature review. GTG, BTW, and TGD wrote the draft manuscript, while SH and TDB reviewed and commented on the draft manuscript. All authors read and approved the final version of the manuscript.

## Conflict of interest

The authors declare that the research was conducted in the absence of any commercial or financial relationships that could be construed as a potential conflict of interest.

## Publisher's note

All claims expressed in this article are solely those of the authors and do not necessarily represent those of their affiliated organizations, or those of the publisher, the editors and the reviewers. Any product that may be evaluated in this article, or claim that may be made by its manufacturer, is not guaranteed or endorsed by the publisher.

## References

[1] Amadou I Lawali S. Smart management of malnutrition using local foods: a sustainable initiative for developing Countries. Front Sustain Food Syst. (2022) 6:1–8. 10.3389/fsufs.2022.725536

[2] Global Panel. The Cost of Malnutrition: Why Policy Action Is Urgent. Global Panel on Agriculture and Food systems for Nutrition. London: Global Panel (2016).

[3] FAO IFAD UNICEF WFP and WHO. Food Security and Nutrition in World: Transforming Food Systems for Food Security, Improved Nutrition and Affordable Healthy Diets for All. Rome: FAO (2021).

[4] Kerac M Blencowe H Grijalva-Eternod C McGrath M Shoham J Cole TJ. Prevalence of wasting among under 6-month-old infants in developing countries and implications of new case definitions using WHO growth standards: A secondary data analysis. Arch Dis Child. (2011) 96:1008–13. 10.1136/adc.2010.191882 21288999 PMC3195296

[5] Oot L Sommerfelt AE Sethuraman K Ross J. Food and Nutrition Technical Assistance III Project: Estimating the Effect of Suboptimal Breastfeeding Practices on Child Mortality?: A Model in Profiles for Country-Level Advocacy. Washington, DC (2015).

[6] Carroll GJ Buccini GS Pérez-Escamilla R. Perspective: what will it cost to scale-up breastfeeding programs? A comparison of current global costing methodologies. Adv Nutr. (2018) 9:572–80. 10.1093/advances/nmy041 30060074 PMC6140429

[7] Bhutta ZA Das JK Rizvi A Gaffey MF Walker N Horton S. Evidence-based interventions for improvement of maternal and child nutrition: what can be done and at what cost? Lancet. (2013) 382:452–77. 10.1016/S0140-6736(13)60996-4 23746776

[8] Haile D Biadgilign S. Higher breastfeeding performance index is associated with lower risk of illness in infants under six months in Ethiopia. Int Breastfeed J [Internet]. (2015) 10:1–7. 10.1186/s13006-015-0057-2 26617666 PMC4662817

[9] WHO and UNICEF. Global Strategy for Infant and Young Child Feeding. Fifthy-Fourth world Health Assembly. (2003).

[10] Sankar MJ Sinha B Chowdhury R Bhandari N Taneja S Martines J. Optimal breastfeeding practices and infant and child mortality: a systematic review and meta-analysis. Acta Paediatr Int J Paediatr. (2015) 104:3–13. 10.1111/apa.13147 26249674

[11] Black RE Victora CG Walker SP Bhutta ZA Christian P De Onis M. Maternal and child undernutrition and overweight in low-income and middle-income countries. Lancet. (2013) 382:427–51. 10.1016/S0140-6736(13)60937-X 23746772

[12] Define breastfeeding, guideline, *Uniceif - Google Search*. Available online at: https://www.google.com/search?q=Define+breastfeeding%2C+guideline%2C+Uniceif&sxsrf=ALiCzsZU1ukJpzmh8TsG3DmCacxDIOZdDA%3A1651470601745&ei=CXFvYtOWLcjekgXinoSAAw&ved=0ahUKEwiTv8u0°j8D3AhVIr6QKHWIPATAQ4dUDCA4&uact=5&oq=Define+breastfeeding%2C+guideline%2C+Uniceif&gs_lcp=Cgdnd3Mtd2l6EAMyBwghEAoQoAEyBwghEAoQoAE6BwgAEEcQsAM6BAgjECc6BQghEKABOggIIRAWEB0QHkoECEEYAEoECEYYAFDXFVjYKWCzK2gBcAF4AIABiAOIAYcTkgEFMi01LjOYAQCgAQHIAQjAAQE&sclient=gws-wiz

[13] Pandolfi E Gesualdo F Rizzo C Carloni E Villani A Concato C. Breastfeeding and respiratory infections in the first 6 months of life: a case control study. Front Pediatr. (2019) 7:1–7. 10.3389/fped.2019.00152 31106183 PMC6492465

[14] Mulugeta SS Muluneh MW Belay AT Moyehodie YA. Multilevel log linear model to estimate the risk factors associated with infant mortality in Ethiopia : further analysis of 2016 EDHS. BMC Pregnancy Childbirth. (2022) 26:597. 10.1186/s12884-022-04868-9 35883058 PMC9316776

[15] Li R Ware J Chen A Nelson JM Kmet JM Parks SE. Breastfeeding and post-perinatal infant deaths in the United States, A national prospective cohort analysis. Lancet Reg Heal - Am. (2022) 5:94. 10.1016/j.lana.2021.100094 35911656 PMC9335131

[16] Abdulla F Hossain MM Karimuzzaman M Ali M Rahman A. Likelihood of infectious diseases due to lack of exclusive breastfeeding among infants in Bangladesh. PLoS ONE. (2022) 17 1–15. 10.1371/journal.pone.0263890 35171952 PMC8849615

[17] Phukan D Ranjan M Dwivedi LK. Impact of timing of breastfeeding initiation on neonatal mortality in India. Int Breastfeed J. (2018) 13:1–10. 10.1186/s13006-018-0162-0 29988694 PMC6029033

[18] Bosnjak AP Grgurić J. Long-term health effects of breastfeeding. Asia Pac J Public Health. (2016) 129:293–8. 10.1177/1010539515624964 18198630

[19] WHO. Acceptable Medical Reasons for Use of Breast-Milk Substitutes. (2009). Available online at: http://www.ncbi.nlm.nih.gov/pubmed/24809113 24809113

[20] UNICEF. Infant and Young Child Feeding. Programing Guide. New York, NY: UNICEF (2012).

[21] Victora CG Bahl R Barros AJD França GVA Horton S Krasevec J. Breastfeeding in the 21st century: epidemiology, mechanisms, and lifelong effect. Lancet [Internet]. (2016) 387:475–90. 10.1016/S0140-6736(15)01024-7 26869575

[22] WHO. Guideline: Protecting, Promoting and Supporting Breastfeeding in Facilities Providing Maternity and Newborn Services. Geneva: World Health Organization (2017). 29565522

[23] Federal Ministry of Health Family Health Department Ethiopia. National Strategy for Infant and Young Child Feeding, Vol. 5. Ethiopia (2004).

[24] Ethiopian Public Health Institute (EPHI) and ICF. Mini Demographic and Health Survey 2019. Rockville, MD: Ethiopian Public Health Institute (EPHI) and ICF (2019).

[25] Monge-Montero C van der Merwe LF Papadimitropoulou K Agostoni C Vitaglione P. Mixed milk feeding: a systematic review and meta-analysis of its prevalence and drivers. Nutr Rev. (2020) 78:914–27. 10.1093/nutrit/nuaa016 32357372

[26] Gómez-Acebo I Lechosa-Muñiz C Paz-Zulueta M Sotos TD Alonso-Molero J Llorca J. Feeding in the first six months of life is associated with the probability of having bronchiolitis: a cohort study in Spain. Int Breastfeed J. (2021) 16:1–11. 10.1186/s13006-021-00422-z 34663376 PMC8522099

[27] Senarath U Dibley MJ Agho KE. Breast-feeding performance index: a composite index to describe overall breast-feeding performance among infants under 6 months of age. Public Health Nutr. (2007) 10:996–1004. 10.1017/S1368980007441428 17381937

[28] Hailu WS Bayih MT Babble NF. Four in every ten infants in Northwest Ethiopia exposed to sub-optimal breastfeeding practice. PLoS ONE. (2020) 15:1–14. 10.1371/journal.pone.0238576 33137137 PMC7605653

[29] Ethiopian Public Health Institute (EPHI) [Ethiopia] ICF. Ethiopia Mini Demographic and Health Survey 2019: Final Report. Rockville, Maryland, USA: EPHI and ICF. (2021).

[30] Roberts TJ Carnahan E Gakidou E. Can breastfeeding promote child health equity? A comprehensive analysis of breastfeeding patterns across the developing world and what we can learn from them. BMC Med. (2013) 11:254. 10.1186/1741-7015-11-254 24305597 PMC3896843

[31] Babaee E Eshrati B Asadi-Aliabadi M Purabdollah M Nojomi M. Early cessation of breastfeeding and determinants: time to event analysis. J Nutr Metab. (2020) 2020:19750. 10.1155/2020/3819750 32399288 PMC7210562

[32] Dagher RK McGovern PM Schold JD Randall XJ. Determinants of breastfeeding initiation and cessation among employed mothers: a prospective cohort study. BMC Pregnancy Childbirth [Internet]. (2016) 16:194. 10.1186/s12884-016-0965-1 27472915 PMC4966748

[33] Alemayehu T Haidar J Habte D. Determinants of exclusive breastfeeding practices in Ethiopia. Ethiop J Heal Dev. (2009) 23:4482. 10.4314/ejhd.v23i1.4483227489561

[34] Mesele HA. Traditional maternal health beliefs and practices in Southern Tigray: the case of raya alamata district. Anat Physiol. (2018) 08:1–12. 10.4172/2161-0940.1000298

[35] Abegunde D Hutchinson P Anaba U Oyedokun-Adebagbo F Johansson EW Feyisetan B. Socioeconomic inequality in exclusive breastfeeding behavior and ideation factors for social behavioral change in three north-western Nigerian states: a cross-sectional study. Int J Equity Health. (2021) 20:1–14. 10.1186/s12939-021-01504-4 34315476 PMC8314581

[36] Tewabe T Mandesh A Gualu T Alem G Mekuria G Zeleke H. Exclusive breastfeeding practice and associated factors among mothers in Motta town, East Gojjam zone, Amhara Regional State, Ethiopia, 2015: a cross-sectional study. Int Breastfeed J. (2017) 12:1–7. 10.1186/s13006-017-0103-3 28261318 PMC5327553

[37] Tang K Wang H Tan SH Xin T Qu X Tang T. Association between maternal education and breast feeding practices in China: a population-based cross-sectional study. BMJ Open. (2019) 9:1–9. 10.1136/bmjopen-2018-028485 31467048 PMC6720234

[38] Kebede EM Seifu B. Breastfeeding and employed mothers in Ethiopia: legal protection, arrangement, and support. Int Breastfeed J. (2021) 16:10–3. 10.1186/s13006-021-00392-2 34127001 PMC8204466

[39] Laksono AD Wulandari RD Ibad M Kusrini I. The effects of mother's education on achieving exclusive breastfeeding in Indonesia. BMC Public Health. (2021) 21:1–6. 10.1186/s12889-020-10018-7 33402139 PMC7786474

[40] Neves PAR Barros AJD Gatica-Domínguez G Vaz JS Baker P Lutter CK. Maternal education and equity in breastfeeding: trends and patterns in 81 low- and middle-income countries between 2000 and 2019. Int J Equity Health. (2021) 20:1–13. 10.1186/s12939-020-01357-3 33413445 PMC7792102

[41] Ayalew T. Exclusive breastfeeding practice and associated factors among first-time mothers in Bahir Dar city, North West Ethiopia, removed: a community based cross sectional study. Heliyon [Internet]. (2020) 6:e04732. 10.1016/j.heliyon.2020.e04732 32944666 PMC7481526

[42] Veeranki SP Nishimura H Krupp K Gowda S Arun A Madhivanan P. Suboptimal breastfeeding practices among women in rural and low-resource settings: a study of women in Rural Mysore, India. Ann Glob Heal [Internet]. (2017) 83:577–83. 10.1016/j.aogh.2017.10.012 29221531

[43] Habtewold TD Sharew NT Alemu SM. Evidence on the effect of gender of newborn, antenatal care and postnatal care on breastfeeding practices in Ethiopia: a meta-Analysis andmeta-regression analysis of observational studies. BMJ Open. (2019) 9:23956. 10.1136/bmjopen-2018-023956 31152028 PMC6549640

[44] Daba D Id J Tenagashaw MW. Breastfeeding practice and factors associated with exclusive breastfeeding among mothers in Horro District, Ethiopia?: A community- based cross-sectional study. PLoS ONE. (2022) 17:e0267269. 10.1371/journal.pone.0267269 35476799 PMC9045649

[45] Woldeamanuel BT. Trends and factors associated to early initiation of breastfeeding, exclusive breastfeeding and duration of breastfeeding in Ethiopia: evidence from the Ethiopia Demographic and Health Survey 2016. Int Breastfeed J. (2020) 15:1–13. 10.1186/s13006-019-0248-3 31924229 PMC6953467

